# Regulation of adipogenic differentiation and adipose tissue inflammation by interferon regulatory factor 3

**DOI:** 10.1038/s41418-021-00798-9

**Published:** 2021-06-05

**Authors:** Peng Tang, Sam Virtue, Jian Yi Gerald Goie, Chin Wen Png, Jing Guo, Ying Li, Huipeng Jiao, Yen Leong Chua, Mark Campbell, José Maria Moreno-Navarrete, Asim Shabbir, José-Manuel Fernández-Real, Stephan Gasser, David Michael Kemeny, Henry Yang, Antonio Vidal-Puig, Yongliang Zhang

**Affiliations:** 1 Department of Microbiology & Immunology, and NUSMED Immunology Translational Research Programme,Yong Loo Lin School of Medicine, National University of Singapore, Singapore, Singapore; 2 Immunology Programme, Life Sciences Institute, National University of Singapore, Singapore, Singapore; 3 Institute of Metabolic Science, Wellcome Trust-MRC MDU Metabolic Disease Unit, University of Cambridge, Addenbrooke’s Hospital, Cambridge, UK; 4 Cancer Science Institute of Singapore, National University of Singapore, Singapore, Singapore; 5 Department of Diabetes, Endocrinology and Nutrition, Institut d’Investigacio Biomedica de Girona (IDIBGI), CIBER Fisiopatologia de la Obesidad y Nutricion (CIBERobn, CB06/03/010), Instituto de Salud Carlos III, and Department of Medical Sciences, Faculty of Medicine, Girona, Spain; 6 Department of Surgery, National University Hospital, Singapore, Singapore

**Keywords:** Interferons, Preclinical research

## Abstract

Dysfunction of adipocytes and adipose tissue is a primary defect in obesity and obesity-associated metabolic diseases. Interferon regulatory factor 3 (IRF3) has been implicated in adipogenesis. However, the role of IRF3 in obesity and obesity-associated disorders remains unclear. Here, we show that IRF3 expression in human adipose tissues is positively associated with insulin sensitivity and negatively associated with type 2 diabetes. In mouse pre-adipocytes, deficiency of IRF3 results in increased expression of PPARγ and PPARγ-mediated adipogenic genes, leading to increased adipogenesis and altered adipocyte functionality. The IRF3 knockout (KO) mice develop obesity, insulin resistance, glucose intolerance, and eventually type 2 diabetes with aging, which is associated with the development of white adipose tissue (WAT) inflammation. Increased macrophage accumulation with M1 phenotype which is due to the loss of IFNβ-mediated IL-10 expression is observed in WAT of the KO mice compared to that in wild-type mice. Bone-marrow reconstitution experiments demonstrate that the nonhematopoietic cells are the primary contributors to the development of obesity and both hematopoietic and nonhematopoietic cells contribute to the development of obesity-related complications in IRF3 KO mice. This study demonstrates that IRF3 regulates the biology of multiple cell types including adipocytes and macrophages to prevent the development of obesity and obesity-related complications and hence, could be a potential target for therapeutic interventions for the prevention and treatment of obesity-associated metabolic disorders.

## Introduction

Obesity is a major contributing factor to the development of chronic medical conditions including type 2 diabetes (T2D), fatty liver, and cardiovascular diseases [1]. This energy balance disorder is characterized by excessive, and often dysfunctional, accumulation of white adipose tissue (WAT) [2]. Adipocytes are the main constituents of WAT, where their primary function is to accommodate energy surplus in the form of triacylglycerol during periods of excessive energy intake and to mobilize it during energy deprivation [3]. In addition, adipocytes produce adipokines such as leptin, resistin, adiponetin and TNFα that modulate systemic metabolism. During the development of obesity, these primary functions of adipocytes are dysregulated, contributing to the development of metabolic disorders including insulin resistance (IR) and T2D [4].

Adipose tissue expansion during obesity can either occur due to an increase in adipocyte number, and/or the size of individual adipocyte in a process known as adipocyte hyperplasia and hypertrophy, respectively. The formation of new adipocytes is known as adipogenesis which includes the commitment of mesenchymal stem cells to pre-adipocytes and the terminal differentiation of pre-adipocytes into insulin-sensitive lipid-filled adipocytes [5]. Adipogenesis is regulated by a number of transcription factors that coordinate the expression of hundreds of genes to establish mature adipocyte phenotype [6]. In response to inductive cues, the transcriptional cascade of adipogenesis starts with the transient expression of two CCAT-enhancer-binding proteins (C/EBP), C/EBPβ and C/EBPδ, which in turn leads to the expression of the lipid-activated nuclear hormone receptor, peroxisome proliferator-activated receptor γ (PPARγ) and C/EBPα [2, 6–8]. PPARγ is both necessary and sufficient for the differentiation of white adipocytes, controlling the entire terminal differentiation process [2, 6–9]. C/EBPα was found to regulate adipogenesis through PPARγ [7, 10]. In addition to these central players, other factors including several KLFs, STAT5, and SREBP-1 promote adipogenesis, whereas GATA2/3, KLF2, HES-1, and TCF/LEF have inhibitory effects [8, 11].

In addition to adipocytes, stromal cells of WAT, macrophages in particular, critically modulate the function of adipose tissues. Obesity induces massive macrophage infiltration and inflammatory activation in WAT causing chronic inflammation [8], a key feature of obesity and T2D [12–14]. Adipose tissue macrophages are a prominent source of pro-inflammatory cytokines including TNFα and IL-6, which can block insulin action [15, 16], causing IR. In obese mice, when inflammatory macrophage infiltration into WAT was inhibited, insulin sensitivity was enhanced [13]. In addition, ablation of GPR105 in mice mitigated high-fat-diet (HFD)-induced IR through inhibition of macrophage recruitment and tissue inflammation [17]. Together, these studies support the causative association of WAT inflammation with IR.

The interferon regulatory factor (IRF) family consists of nine transcription factor proteins that play important and diverse functions in regulation of cellular responses [18, 19]. Among them, IRF4 positively regulates lipolysis and suppress lipogenesis in WAT [20], and plays a central role in adaptive thermogenesis [21]. IRF3 is expressed in 3T3-L1 pre-adipocytes and regulates its differentiation to adipocytes [22]. Here we show the roles of IRF3 in adipogenesis, adipocyte function, and WAT macrophage activation, which are important for maintaining metabolic homeostasis.

## Materials and methods

### Animal experiments

Animal experiments were approved by the Institutional Animal Care and Use Committee of National University of Singapore or the University of Cambridge License Review Panel and approved by the UK Home Office. The IRF3 deficient mice [23] in C57BL/6 background and the C57BL/6 mice were maintained under a 12 h light/12 h dark cycle at constant temperature (23 °C) with free access to food and water. Both wild-type (WT) and knockout (KO) mice were randomized to different treatment groups. The investigators were aware of the genotypes of the animals used. No statistical methods were used to pre-determine sample sizes. The insulin tolerance test (ITT) was performed on mice fasted for 2 h by intraperitoneal injection of insulin at 0.75 U/Kg (insulin/body weight). For the glucose tolerance test (GTT), mice were fasted overnight before administration of 2 g/kg d-glucose into the peritoneum. Blood glucose was measured before and after the insulin or glucose administration using Accu-Chek® Advantage glucose meter and test strips (Roche).

### Indirect calorimetry

Animals were placed in Metatrace system for 48 h (Creative Scientific, UK) at 21 °C. Airflow rates were 400 mL/min and measurements of oxygen concentration and carbon dioxide concentration in room air and air leaving each cage were taken every 10 min. The activity was assessed by beam breaks that were 2.5 cm apart. Food intake and water were weighed into and out of the system.

### Human adipose tissue

Twenty-eight subcutaneous adipose tissue samples from a group of morbidly obese (BMI > 35 kg/m^2^) caucasian subjects with different degrees of insulin action [measured using hyperinsulinemic-euglycemic clamp [24]] recruited at the Endocrinology Service of the Hospital Universitari Dr. Josep Trueta (Girona, Spain) were studied. All subjects reviewed that their body weight had been stable for at least 3 months. They had no systemic disease other than obesity and were all free of any infections in the previous month before the study. Liver disease and thyroid dysfunction were specifically excluded by biochemical workup. All subjects gave written informed consent after the purpose, nature and potential risks for the study were explained to them. The Hospital Ethics Committee approved the protocol. Adipose tissue samples were obtained during elective surgical procedures (cholecystectomy, surgery of abdominal hernia, and gastric by-pass surgery), washed, fragmented, and immediately flash-frozen in liquid nitrogen before being stored at −80 °C.

RNA purification and gene expression procedures and analyses were performed as previously described [24]. Briefly, RNA purification was performed using RNeasy Lipid Tissue Mini Kit (QIAgen, Izasa SA, Barcelona, Spain) and the integrity was checked by Agilent Bioanalyzer (Agilent Technologies, Palo Alto, CA). Gene expression was assessed by real-time PCR using an LightCycler® 480 Real-Time PCR System (Roche Diagnostics SL, Barcelona, Spain), using TaqMan® technology suitable for relative genetic expression quantification. The commercially available and pre-validated TaqMan® primer/probe sets used for gene expression analyses (from Life technologies): IRF3 (Hs01547282_m1), IRS1 (Hs00178563_m1), GLUT4 or SLC2A4 (Hs00168966_m1), PPIA (Housekeeping. Hs99999904_m1).

Glycosylated hemoglobin (HbA1c) was measured by the high-performance liquid chromatography method (Bio-Rad, Muenchen, Germany, and autoanalyser Jokoh HS-10, respectively). Intra- and inter-assay coefficients of variation were less than 4% for all these tests.

### Cell culture and differentiation

3T3-L1 cells free of mycoplasma were purchased from ATCC and were maintained in Dulbecco’s modified Eagle’s medium (DMEM) supplemented with 10% calf serum (Invitrogen). Differentiation was induced by treatment with 100 μg/mL insulin, 1.15 mg/mL 3-isobutyl-1-methylxanthine, and 1μM dexamethasone in DMEM supplemented with 10% fetal bovine serum (FBS). Two days after the treatment, cells were maintained in DMEM supplemented with 10% FBS and 100 μg/mL insulin. Cells were maintained in the medium for additional 8 days with the media changed every other day.

For isolation of adipose tissue macrophages, adipocytes, and stromal vascular cells (SVCs), adipose tissues isolated from mice were minced into fine pieces immediately after CO_2_ asphyxiation and were digested in HEPES-buffered DMEM supplemented with 2.5% BSA and 40 μg/mL collagenase at 37 °C on an orbital shaker (200 rpm) for 45–60 min. Once digestion was complete, the samples were passed through a sterile 100-μm nylon mesh and the suspension was placed on ice for 20 min. The upper white fat layer was collected as mature adipocytes. The remaining suspension was centrifuged at 1000 rpm for 5 min and the pelleted cells were collected as the SVCs.

To isolate mouse pre-adipocytes for adipocyte differentiation, WATs isolated from mice at the age of 6 weeks were minced into fine pieces immediately after CO_2_ asphyxiation and were digested in Hanks’ Balanced Salt Solution containing 1 mg/mL collagenase type II at 37 °C on an orbital shaker (200 rpm) for 30–40 min, followed by filtering through a sterile 100-μm nylon mesh. The solution was collected in a 50 mL tube and the tube was placed on ice for 20 min. The floating adipocyte fraction was removed and the supernatant was transferred into a new tube and mixed with DMEM (high glucose) medium containing 10% newborn calf serum (NCS) at 1:1 ratio, followed by centrifugation at 700 × *g* for 10 min. The cell pellet was re-suspended in DMEM (high glucose) medium containing 10% NCS plus pen/strep, l-glutamine, 24 nM insulin, and 150 μm sodium ascorbate, and was plated onto 12-well culture plates. The medium was changed every 2 days until the end of the differentiation.

### Immunophenotyping and Flow cytometry

The erythrocyte-depleted SVCs isolated from adipose tissue were incubated with fluorophore-conjugated antibodies including CD11b-phycoerythrin (CD11b-PE), CD45.2-PE (eBioscience), and F4/80-APC (Caltag Laboratories Inc.) or isotype control antibodies for 30 min on ice followed by washing with FACS buffer twice. Cells were resuspended in FACS buffer and analyzed on a FACS Calibur. Data analysis was performed using FlowJo software (Tree Star, Ashland, OR).

### Biochemical analysis

Serum insulin levels were measured by a mouse insulin ELISA kit (Millipore). Serum adiponectin was determined by a mouse adiponectin ELISA kit (Millipore). Serum triglycerides were determined by a kit from Sigma. Serum NEFAs were measured using NEFA C kit (Wako Chemical, Richmond, VA).

### Immunohistochemical and morphometric analyses

Adipose tissues were isolated and fixed overnight in 4% paraformaldehyde and embedded in paraffin. Five-micrometer sections were cut and stained with hematoxylin and eosin. To determine the size of adipocytes, diameters of individual cells were measured digitally in histological light-microscopic images [10×] of adipose tissue cross-sectional areas using ImageJ software (NIH, Bethesda, MD). At least 300 individual cells (*n* = 100 adipocytes/section, 1 section/animal, 3 animals/group) from randomly selected areas in histological sections in both WT and KO WATs were measured. Paraffin sections of perigonadal WAT were stained with anti-mouse F4/80 antibody (eBioscience).

### Analysis of gene expression by qPCR and microarray

Tissues were snap-frozen in liquid nitrogen and total RNA was isolated using NucleoSpin® RNA kit (Macherey-Nagel) for cDNA synthesis using reverse transcriptase and oligo (dT) primers (Promega). SYBR green-based quantitative PCR (qPCR) was performed using *Gapdh* as a housekeeping gene control.

For microarray gene expression, total RNA was extracted from adipocytes harvested on various days during differentiation with TRIzol reagent (Invitrogen). RNA integrity was confirmed by nondenaturing agarose gel electrophoresis. RNA from each sample was further purified to remove contaminating organics and non-RNA species using a silica resin (RNeasy; Qiagen) according to the manufacturer’s instructions. Each RNA sample was labeled using Illumina TotalPrep‐96 RNA Amplification kit (Lot # 1305035, Ambion) as per amplification protocol. One thousand five hundred nanograms of the generated cRNA was hybridized onto a Mouse Ref‐8 V2 BeadChip. The BeadChip was incubated at 58 °C, with rotation speed 5 for 18 h for hybridization. The BeadChip was washed and stained as per Illumina protocol and scanned on the iScan (Illumina). The data files were quantified in GenomeStudio Version 2011.1 (Illumina).

### Protein extraction and western blot analysis

Protein was extracted from tissues including WAT, skeletal muscle, and the liver using Triton lysis buffer containing protease and phosphatase inhibitors. Protein was subjected to SDS-Page and transferred to nitrocellulose followed by probing with appropriate antibodies including anti-phospho-IRF3 (Cell Signaling), anti-IRF3 (Santa Cruz Biotech), anti-phospho-AKT (Cell Signaling), anti-AKT (Cell Signaling), anti-PPARγ (Cell Signaling) antibodies.

### Statistical analysis

Sample sizes were determined based on the amount of data required to give the statistical significance. No data were excluded from the study. Data are presented as mean value + standard error of the mean (SEM). Statistical differences between groups were analyzed using two tailed Student’s *t* test. We used individual glucose values in ITT and GTT assays at each time point for statistical analysis. Values of *P* < 0.05 were defined as statistically significant.

## Results

### Association of IRF3 expression with obesity

To understand the role of IRF3 in metabolism, we fed C57BL/6 mice with a HFD for 8 and 16 weeks to examine the expression of IRF3. After HFD for 8 weeks, we observed a significant increase in mRNA expression of IRF3 in WAT, but not in skeletal muscle or the liver, compared to that in mice fed with chow (Supplementary Fig. 1a). We also observed increased IRF3 protein expression and phosphorylation in WAT from HFD-fed mice compared with chow-fed mice (Supplementary Fig. 1b). Interestingly, IRF3 expression in WAT was lower than that in skeletal muscle or the liver with or without HFD for 16 weeks (Supplementary Fig. 1C). Furthermore, after 16 weeks of HFD, reduced IRF3 expression in both visceral WAT (vWAT, also known as perigonadal WAT) and subcutaneous WAT (sWAT) compared to that in chow-fed mice was observed (Supplementary Fig. 1C), whereas IRF3 activation was undetectable. These results suggest that the development of severe obesity in mice is associated with the loss of IRF3 function in WAT.

**Fig. 1:**
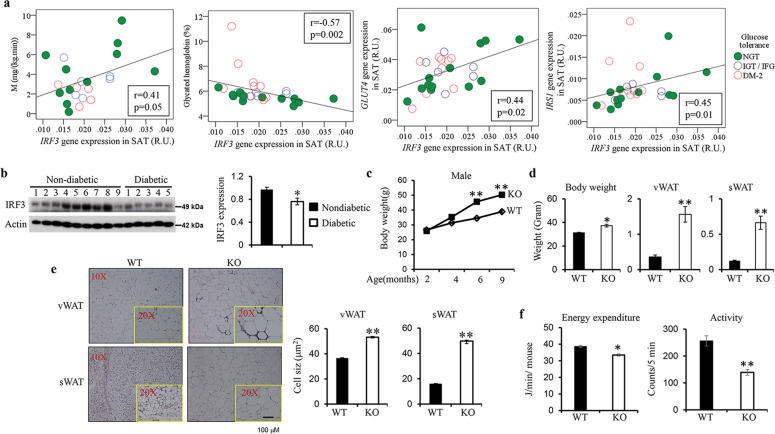
Negative association of IRF3 expression with type 2 diabetes in humans and development of obesity in IRF3 deficient mice. **a** Association of IRF3 in subcutaneous adipose tissue from human morbid obese subject (no other disease) with insulin sensitivity is determined by euglycemic clamp. IRF3 expression in both subcutaneous and visceral adipose tissue is negatively associated with glycated hemoglobin and is positively associated with GLUT4 and IRS1 expression. (NGT: normal glucose tolerance; IGT/IFG: impaired glucose tolerance/impaired fasting glucose; DM-2: type 2 diabetes.). **b** IRF3 expression in visceral adipose tissue from obese nondiabetic and obese diabetic patients was examined by western blot analysis. **c** Growth curve of wild-type (WT) and IRF3 knockout (KO) male mice on chow diet was determined. **d** Body weight, weight of visceral WAT (vWAT), and subcutaneous WAT (sWAT) from WT and IRF3 KO male mice at the age of 4 months. **e** Representative sections of vWAT and sWAT from WT and IRF3 KO mice at the age of 4 months stained with H&E. Sizes of adipocytes were measured digitally with ImageJ software and the average size of 300 cells of both WT and IRF3 KO were presented. The data shown are representative of three independent experiments with similar results. Data are presented as mean ± SEM. *p < 0.05, **p < 0.01. **f** Energy expenditure and activity of WT and IRF3 KO male mice at the age of 6 months were analyzed by Oxymax/CLAMS (Columbus Instruments).

The expression of *IRF3* in subcutaneous adipose tissues of human morbid obese subjects was shown to be positively correlated with peripheral glucose disposal (Fig. 1a) and was negatively correlated with glycated hemoglobin, an indicator of glycemic control and a clinical marker of the severity of diabetes [25]. Furthermore, *IRF3* expression was also positively associated with the expression of *IRS1* and *GLUT4*, two markers of insulin action. In addition, reduced IRF3 protein expression was observed in visceral adipose tissue from obese with T2D patients compared with that from obese but nondiabetic patients (Fig. 1b). These results suggest a regulatory function of IRF3 in obesity and obesity-associated metabolic disorders.

### Development of obesity in IRF3 deficient mice

To examine the role of IRF3 in obesity, we compared the body weight of gender- and age-matched WT and IRF3 KO mice at various ages. From the age of 4 months onward, the weight of IRF3 KO male mice was significantly greater than WT mice on normal chow (Fig. 1c). There was also a significant increase in body weight of female KO mice at the age of 6 months compared with age-matched WT female mice (Supplementary Fig. 1d). In addition, KO male mice at the age of 4 months and female mice at the age of 6 months had larger subcutaneous and perigonadal fat pads than age- and gender-matched WT littermates (Fig. 1d and Supplementary Fig. 1e, f). Histological analysis of WAT cross-sections revealed increased size of adipocytes in both vWAT and sWAT from KO mice compared to WT (Fig. 1e). These results indicate that deficiency of IRF3 results in the development of obesity with aging.

Monitoring of food intake by WT and KO mice at the age of 3–4 months showed comparable food intake (Supplementary Fig. 1g). Analysis of metabolic rates of the mice showed that the KO mice had reduced energy expenditure, which, when corrected for body weight, was around 15% lower than WT mice (Fig. 1f and Supplementary Fig. 1h). In addition, the KO animals exhibited reduced activity compared to WT (Fig. 1f). These results suggest that IRF3 KO mice are hypometabolic, with reduced activity and energy expenditure.

### Impaired glucose homeostasis by the absence of IRF3

**Fig. 2:**
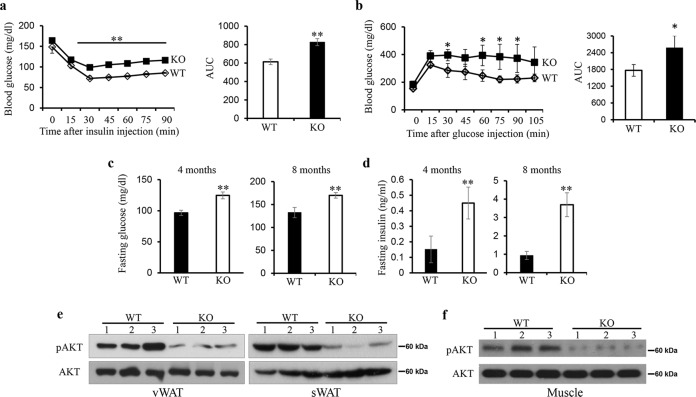
IRF3 deficiency resulted in impaired glucose homeostasis and type 2 diabetes. **a** Insulin tolerance tests were performed in sex-matched 5-month-old WT (*n* = 5) and IRF3 KO mice (*n* = 5) after injection with 0.7 U/kg human insulin (Sigma, MO). **b** Glucose tolerance tests were performed on sex-matched 5-month-old WT (*n* = 5) and IRF3 KO mice (*n* = 5) after overnight fasting by giving 2 g/kg of d-glucose i.p. Blood samples were taken at the indicated time points to measure blood glucose levels. The data shown are representative of five independent experiments with similar results. AUC area under curve. **c** Fasting blood glucose levels of 4 and 8-month-old mice were determined. **d** Fasting serum insulin levels were determined on mice at the age of 4 and 8 months. Protein expression and phosphorylation of AKT in adipose tissues (**e**) and skeletal muscle (**f**) from WT and KO mice (nonfasting condition) were detected by western blot. The data shown are representative of three independent experiments with similar results. Data are presented as mean ± SEM. *p < 0.05, **p <  0.01.

Next, an ITT and GTT were performed on WT and KO mice and found that both male and female KO mice developed IR and glucose intolerance around the age of 5 months compared to WT mice (Fig. 2a, b and Supplementary Fig. 2a). We also detected a significant increase in fasting blood glucose and insulin levels in male mice at the age of 4 months (Fig. 2c, d). Around the age of 8 months, both male and female KO mice had increased fasting glucose levels of around 180 mg/dl, and increased fasting serum insulin levels as compared with WT mice (Fig. 2c, d and Supplementary Fig. 2b), indicating that IRF3 KO mice progressively develop hyperglycemia and hyperinsulinemia. Consistently, decreased AKT phosphorylation in both vWAT and sWAT and in skeletal muscle from IRF3 KO mice was observed (Fig. 2e, f). Therefore, IRF3 deficiency results in impaired glucose homeostasis and insulin sensitivity, which eventually leads to the development of diabetes around the age of 8 months.

### IRF3 inhibits PPARγ expression and adipogensis

**Fig. 3:**
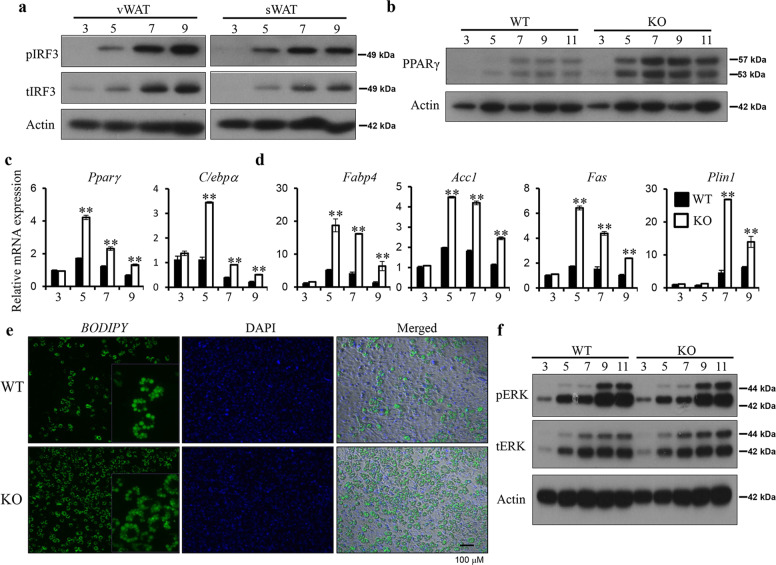
IRF3 negatively regulates adipocyte differentiation. **a** Pre-adipocytes were isolated from vWAT and sWAT of WT mice and were harvested at the indicated days during differentiation. IRF3 expression and phosphorylation were determined by western blot analysis. **b** PPARγ protein expression in WT and IRF3 KO adipocytes at various days during differentiation was determined by western blot analysis. The expression of PPARγ and C/EBPα (**c**) as well as several PPARγ-targeted genes indicated (**d**) at various days during adipocyte differentiation from pro-adipocytes were determined by qPCR. **e** WT and IRF3 KO adipocytes on day 11 after differentiation were stained with *BODIPY* 493/503. **f** ERK phosphorylation in WT and IRF3 KO adipocytes at various days during differentiation was examined by western blot analysis. The data shown are representative of three independent experiments with similar results. Data are presented as mean ± SEM. *p < 0.05, **p < 0.01.

To examine the role of IRF3 in adipogenesis, pre-adipocytes from WT and KO mice were isolated from both vWAT and sWAT for adipocyte differentiation. IRF3 expression and activation were found to be induced on day 5 after initiation of differentiation and greatly increased on days 7–9 (Fig. 3a). Interestingly, the expression of PPARγ was also induced on day 5 and increased on days 7–9 in both WT and KO cells (Fig. 3b). However, when compared to WT cells, greatly increased expression of both mRNA and protein of PPARγ in KO cells was observed (Fig. 3b, c). mRNA expression of C/EBPα was also increased between days 5 and 9 in KO cells (Fig. 3c). Interestingly, the expression of C/EBPβ and C/EBPδ was not changed (Supplementary Fig. 3a), suggesting that IRF3 directly regulates PPARγ or regulates PPARγ through factors downstream of C/EBPβ and C/EBPδ. Consequently, the expression of lipogenic genes including *aP2/Fabp4, Acc1, Fas*, and *Plin1* was increased in KO cells compared to WT cells (Fig. 3d). In line with the increased expression of adipogenic genes, more lipid droplets were observed in KO cells compared to WT cells (Fig. 3e), whereas the number of WT and KO cells was comparable on day 11 after differentiation (Supplementary Fig. 3b). The expression and activation of ERK, an important regulator of cell proliferation, were also comparable between WT and KO cells during differentiation (Fig. 3f). Together, these results indicate that IRF3 regulates adipocyte differentiation, but not proliferation, during adipogenesis.

### IRF3 suppresses PPARγ-mediated transcriptional program during adipocyte differentiation

**Fig. 4:**
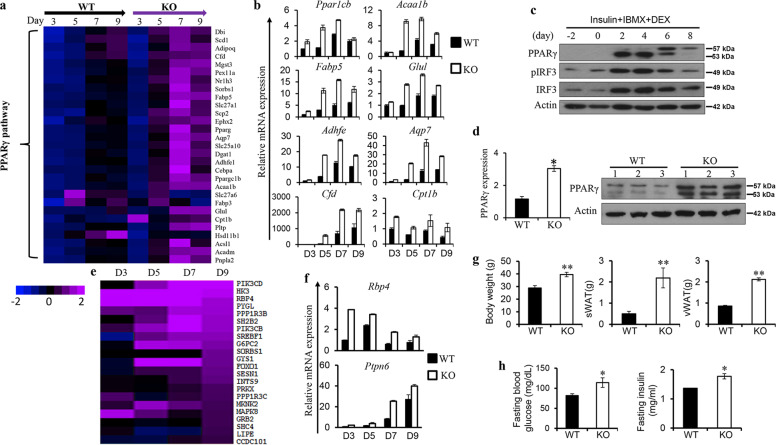
IRF3 regulates transcriptional network of adipocyte differentiation and adipocyte functionality. WT and IRF3 KO pre-adipocytes were isolated from WAT and were used for differentiation. Cells were harvested for RNA at various days indicated during differentiation for microarray analysis. **a** Heat map showing adipogenesis genes in WT and KO cells during differentiation. **b** WT and IRF3 KO cells harvested at indicated days during differentiation were used for cDNA. The expression of PPARγ-targeted genes was determined by qPCR. **c** Expression of PPARγ, expression, and phosphorylation of IRF3 at indicated time points during 3T3-L1 adipocyte differentiation were analyzed by western blot. **d** mRNA and protein expression of PPARγ in WAT from WT and IRF3 KO mice at the age of 9 months. The data shown are representative of three independent experiments with similar results. **e** Heat map showing enriched expression of insulin-resistant genes in IRF3 KO adipocytes during differentiation. **f** Expression of RBP4 and PTPN6 in WT and KO adipocytes during differentiation was determined by qPCR. **g**, **h** WT and IRF3 KO male mice at the age of 8 weeks were lethally irradiated followed by transferring with WT bone-marrow cells. Eighteen weeks after bone-marrow reconstitution, weight of body, vWAT and sWAT of the mice were determined **g** Fasting blood glucose and insulin levels were analysed (**h**). Data are presented as mean ± SEM. *p < 0.05, **p < 0.01.

Microarray analysis of WT and IRF3 KO cells at different time points during differentiation demonstrated a sequential upregulation of adipogenesis-related genes between days 3 and 9 in WT cells (Supplementary Fig. 3c; GEO No.: GSE172491). A similar upregulation of these genes was observed in KO cells. However, compared with WT cells, KO cells demonstrated an earlier upregulation of these genes, especially between days 3 and 5 (Fig. 4a and Supplementary Fig. 3d).

We next analyzed the expression of PPARγ-targeted genes. In both WT and KO cells, sequential upregulation of PPARγ, C/EBPα, and PPARγ-targeted genes including *Adipoq*, *Fabp5*, *Dgat1*, *Acaa1b*, *Cpt1b*, and *Glul* was observed from day 3 to day 9 during differentiation (Supplementary Fig. 3e). Most of these PPARγ-targeted genes in KO cells exhibited earlier and increased expression at all the time points examined compared to WT cells (Fig. 4a and Supplementary Fig. 3e), which were confirmed by quantitative real-time PCR (qPCR) (Fig. 4b). Together, these results demonstrate the negative regulation of PPARγ-targeted adipogenesis program by IRF3.

Next, we examined IRF3 and PPARγ expression in 3T3-L1, a pre-adipocyte cell line, to understand the interaction between these two proteins in adipogenesis. Low levels of IRF3 protein expression and phosphorylation, but not the expression of PPARγ, was detected without adipogenic stimulation (Fig. 4c). Upon exposure to differentiation stimuli (insulin + IBMX + DEX), PPARγ expression was greatly induced on day 2, sustained on day 4, and subsequently reduced on day 6 onward (Fig. 4c). Meanwhile, the expression and activation of IRF3 were also greatly increased on day 2 and day 4, followed by reduction on day 6. In addition, increased expression of PPARγ in KO WAT compared to that in WT WAT was observed (Fig. 4d), suggesting that IRF3 negatively regulates PPARγ expression.

### Altered functionality of IRF3 KO adipocytes

In addition to PPARγ-regulated adipogenesis genes, enriched expression of genes involved in TNFα receptor signaling, cytokine and cytokine receptor signaling, and PKCδ/PTPN6 pathway was observed in KO adipocytes compared to WT cells (Supplementary Fig. 4a, b, c). Interestingly, a group of genes that are dysregulated in TNFα-induced IR 3T3-L1 adipocytes [26] was found to be overrepresented in IRF3 KO adipocytes (Fig. 4e). Among them, *Rbp4*is causatively linked with IR [27]. Increased *Rbp4* expression in KO adipocytes compared to WT cells was confirmed by qPCR (Fig. 4f). In addition, increased expression of PTPN6, a molecule that negatively modulates glucose homeostasis [28], in KO cells was also detected (Fig. 4f). Furthermore, increased expression of various chemokines including *Ccl3, Ccl6*, and *Ccl9* was detected in KO adipocytes compared to WT cells (Supplementary Fig. 4d), indicating enhanced ability of KO adipocytes to recruit immune cells, especially monocytes/macrophages to adipose tissue. Together, these results indicate the aberrant function of IRF3 KO adipocytes.

To investigate the contribution of nonhematopoietic cells such as adipocytes to the development of obesity and T2D in IRF3 KO mice, we lethally irradiated WT and IRF3 KO mice followed by transferring WT bone-marrow (WT-BM) cells. Interestingly, we observed the development of obesity in irradiated KO mice received WT-BM cells (KO chimera) compared to irradiated WT mice received WT-BM cells (WT chimera) (Fig. 4g and Supplementary Fig. 4e). In addition, increased fasting blood glucose and insulin levels were observed in KO chimera compared to WT chimera (Fig. 4h), indicating the development of hyperglycemia and hyperinsulinemia in the KO chimera. Together, these results suggest that deficiency of IRF3 resulted in altered adipogenesis and adipocyte functionality which promote the development of metabolic disorders.

### Increased inflammation and macrophage infiltration in IRF3 KO WAT

**Fig. 5:**
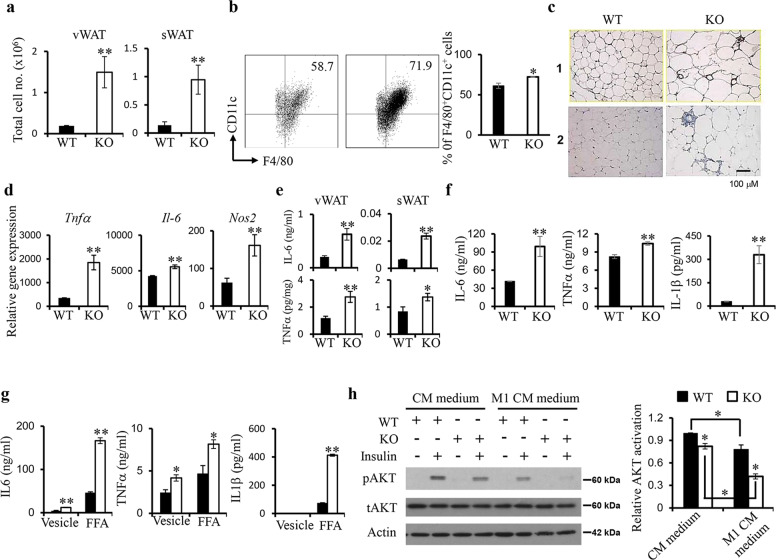
Increased inflammatory macrophage infiltration in IRF3 KO WAT and IRF3 KO macrophages impair adipocyte insulin sensitivity. **a** Total infiltrated cell numbers in stromal vascular fraction (SVF) of WAT from WT and IRF3 KO mice at the age of 5 months. **b** WT and KO SVF cells were stained with antibodies against CD45.2, F4/80, and CD11c and analyzed by flow cytometry. Live gated CD45.2^+^ cells were analyzed for F4/80 and CD11c expression. **c** F4/80 staining of sections of vWAT from WT and IRF3 KO mice at the age of 4 months. Results from 2 WT and 2 KO mice were shown. **d** vWAT was taken from 6-month-old WT and IRF3 KO male mice to isolate SVF cells for gene expression analysis by qPCR. **e** vWAT and sWAT were taken from 6-month-old WT and IRF3 KO male mice. 0.3 g of tissue slices from each were cultured in PRMI medium overnight to determine IL-6 and TNFα production. **f** WT and KO BMDMs were stimulated with M1 inducer (100 ng/mL LPS and 20 ng/mL IFNγ) overnight. The concentration of IL-6, TNFα, and IL-1β in culture supernatants was determined. **g** WT and KO BMDMs were stimulated with or without 500 μM palmitic acid (FFA) overnight to examine cytokine production. **h** Differentiated 3T3-L1 adipocytes were pre-treated with culture supernatants (conditioned medium, CM) of WT and KO BMDMs with or without M1 activation overnight followed by simulation with or without 100 nM insulin for 15 min. AKT phosphorylation was determined. The bar chart showing the relative AKT phosphorylation. The data shown are representative of three independent experiments with similar results. *p < 0.05, **p < 0.01.

WAT macrophage infiltration is primarily responsible for obesity-associated inflammation [12, 14]. The increased number of infiltrated cells in KO WAT compared to WT was observed (Fig. 5a). The percentage of F4/80^+^CD11c^+^ macrophages, known as M1 macrophages in WAT [16], is increased in the stromal vascular fraction (SVF) of KO WAT compared to WT WAT (Fig. 5b). KO vWAT sections stained with anti-F4/80 antibody showed an increase of F4/80^+^ cells surrounding adipocytes, forming a typical crown-like structure (Fig. 5c). Significantly increased expression of inflammatory genes including *Tnf*α, *Il6,* and *Nos2* was observed in SVF from KO vWAT (Fig. 5d). In addition, both KO sWAT and vWAT explants secreted increased amount of IL-6 and TNFα compared to WT samples (Fig. 5e). Interestingly, the expression of M2 markers including *Mgl1*, *Mgl2*, *Mrc2,* and *Chi3l3* were also increased in KO SVF compared to that in WT SVF (Supplementary Fig. 5a). When cultured in M1 or M2 conditions, KO BM-derived macrophages (BMDMs) had increased expression of M1 or M2 markers respectively compared to WT BMDMs (Supplementary Fig. 5b, c). Furthermore, we detected increased production of TNFα, IL-6 and IL-1β by KO BMDMs compared to WT cells upon M1 activation (Fig. 5f). Similarly, KO BMDMs produced increased amount of TNFα, IL-6 and IL-1β in response to FFA (Fig. 5g).

Next, culture supernatants (conditioned medium (CM)) from WT and KO BMDMs with or without M1 activation were incubated with differentiated 3T3-L1 adipocytes followed by insulin stimulation. Adipocytes incubated with M1-activated WT or KO CM had reduced AKT activation upon insulin stimulation, but KO CM caused a more severe impairment of AKT activation than WT CM (Fig. 5h). In addition, IRF3 KO macrophages cocultured with adipocytes resulted in impaired AKT activation in response to insulin compared to WT macrophages cocultured with adipocytes (Supplementary Fig. 6a). Together, these results demonstrate that the development of obesity in IRF3 KO mice is accompanied by increased M1 macrophage infiltration and WAT inflammation, which contributed to impaired insulin sensitivity. In line with these observations, KO WATs showed an enrichment of phagocytic-related genes compared to WT WAT (Supplementary Fig. 6b).

### IRF3 inhibits macrophage inflammatory activation through IFNβ-induced IL-10

**Fig. 6:**
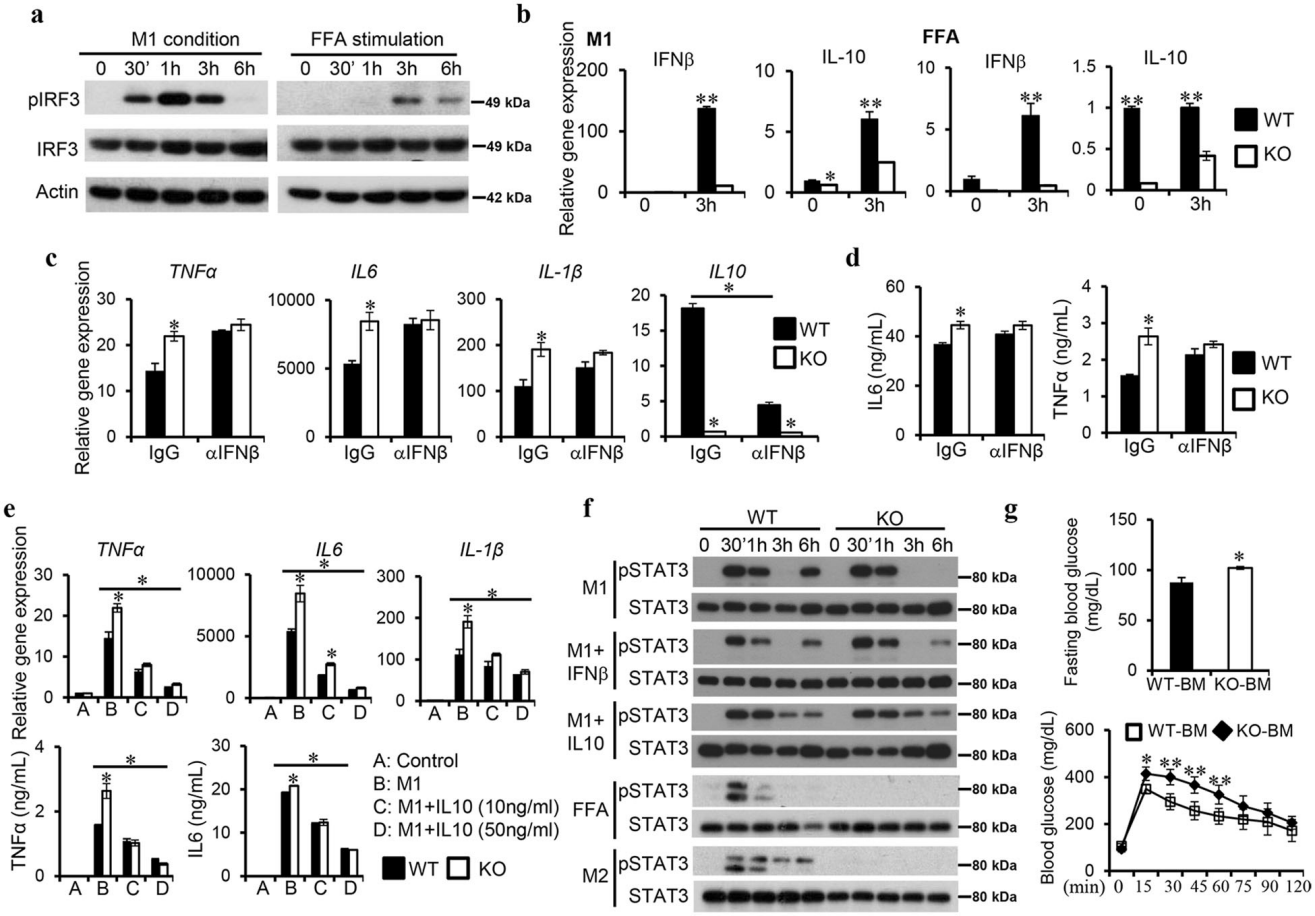
IRF3 inhibits macrophage inflammatory activation and adipose tissue inflammation through IFNβ/IL-10 axis. **a** WT BMDMs were stimulated with M1 activator or 500 μM FFA to examine IRF3 activation by western blot. **b** IFNβ and IL-10 expression in WT and KO BMDMs in response to M1 or FFA stimulation was determined by qPCR. **c**, **d** WT and KO BMDMs were treated with IFNβ neutralizing antibody or IgG control followed by M1 stimulation. Cytokine expression was determined by qPCR (**c**) or ELISA (**d**). **e** WT and KO BMDMs were activated with M1 inducers with or without IL-10. Cytokine expression was determined by qPCR. **f** WT and KO BMDMs were treated with M1, M1 plus IFNβ (2 ng/mL), M2, or FFA to examine STAT3 activation by western blot. The data shown are representative of three independent experiments with similar results. **g** Lethally irradiated WT mice were transferred WT (WT-BM) or IRF3 KO BM (KO-BM) cells. Three months after reconstitution, fasting blood glucose levels were determined. Glucose tolerance tests were performed. Data are presented as mean ± SEM. *p < 0.05, **p < 0.01.

IRF3 activation in macrophages was found to be induced by both M1 and FFA stimulation (Fig. 6a). Interestingly, PPARγ expression was reduced in KO cells compared to that in WT cells upon M1 stimulation (Supplementary Fig. 6c). To examine if IRF3 exerts its anti-inflammatory function through type I IFNs, WT, and KO BMDMs were subjected to M1 or FFA stimulation. Impaired expression of IFNβ in KO cells was observed (Fig. 6b). The expression of IL-10, a potent anti-inflammatory cytokine, in KO cells was also reduced. Pre-treatment of cells with an IFNβ neutralizing antibody increased the expression of TNFα, IL-6, and IL-β in WT cells to levels comparable to these in KO cells in response to M1 activation, which is correlated with a great reduction of IL-10 expression (Fig. 6c). Similarly, IFNβ neutralization resulted in comparable secretion of IL-6 and TNFα between WT and KO macrophages upon M1 stimulation (Fig. 6d). These results suggest that IRF3 inhibits macrophage inflammatory cytokine expression through IFNβ.

To understanding the contribution of IL-10 to the anti-inflammatory function of IRF3, WT and KO macrophages were treated with M1 activators with or without exogenous IL-10. IL-10 treatment resulted in reduced mRNA expression of TNFα, IL-6, and IL-β in both WT and KO cells in a dose-dependent manner. Importantly, the addition of IL-10 diminished the difference on the expression of those cytokines between WT and KO cells (Fig. 6e). Similar results were observed in protein expression of TNFα and IL-6 (Fig. 6e).

IL-10 signals through STAT3 to dampen inflammation [29]. We detected impaired STAT3 phosphorylation in IRF3 KO macrophages at 6 h post-M1 stimulation (Fig. 6f). Exogenous IFNβ or IL-10 restores later STAT3 phosphorylation in KO macrophages. Impaired STAT3 phosphorylation in KO macrophages in response to FFA or M2 stimulation was also observed (Fig. 6f). On the other hand, comparable STAT3 phosphorylation was detected between WT and KO cells in response to IFNβ or IL-10 stimulation (Supplementary Fig. 7a). In addition, IFNβ was able to induce higher IL-10 levels in KO macrophages compared to WT cells (Supplementary Fig. 7b), indicating that deficiency of IRF3 does not impair interferon receptor or STAT3 signaling. Together, these results demonstrate that IRF3 restrains obesity-associated macrophage inflammatory activation through IFNβ-induced IL-10.

To investigate the contribution of hematopoietic cells such as macrophages to the development of obesity and T2D in IRF3 KO mice, we transferred WT- or KO-BM cells to lethally irradiated WT mice. We observed comparable weights of body and WAT between mice received WT- or KO-BM cells (Supplementary Fig. 7c, d). However, compared to mice received WT-BM cells, increased fasting blood glucose levels and glucose intolerance were observed in mice received KO-BM cells (Fig. 6g). Therefore, the nonhematopoietic compartment in IRF3 KO mice is primary responsible for the development of obesity (Fig. 4g and Supplementary Fig. 4e), whereas both the nonhematopoietic and hematopoietic compartments contribute to the development of the impaired glucose tolerance.

## Discussion

Adipocyte differentiation and adipocyte hypertrophy are the two key processes in determining fat mass under energy excessive condition. Differentiation from pre-adipocytes to mature adipocytes is controlled by a PPARγ-mediated transcriptional network. Here, we show that IRF3 is critical for controlling this process by acting as a brake to prevent hypertrophic obesity, an underlying pathological alteration linked to the development of obesity-associated diseases including IR and T2D [30]. In humans, we found that the expression of IRF3 in WAT is positively correlated with peripheral insulin sensitivity and is negatively correlated with severity of diabetes (Fig. 1a, b). In addition, reduced IRF3 protein expression was observed in WAT from obese T2D patients compared with that in obese patients without T2D (Fig. 1c). There findings support a protective role of IRF3 in the development of obesity-associated metabolic abnormalities.

**Fig. 7:**
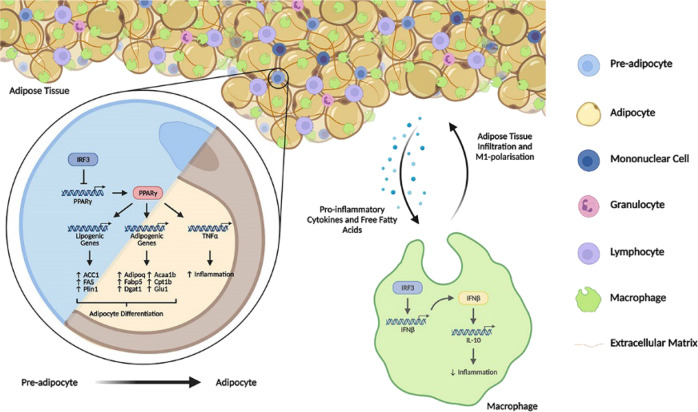
Protective function of IRF3 in development of obesity and adipose tissue inflammation. In pre-adipocytes, in response to differentiation cues, IRF3 inhibits the transcription and expression of PPARγ, a master regulator of adipogenesis, thereby controlling the transcription and expression of lipogenic and adipogenic genes and suppressing the differentiation of pre-adipocytes to adipocytes. The loss of IRF3 leads to uncontrolled adipocyte differentiation and increased production of pro-inflammatory cytokines such as TNFα. Such pro-inflammatory cytokines coupled with free fatty acids promote macrophage infiltration into adipose tissues and subsequent M1-polarization. In addition, the loss of IRF3 in macrophages impairs the expression of IFNβ and IFNβ-mediated IL-10 production. The loss of this anti-inflammation axis contributes to chronic inflammation and the development of obesity-mediated metabolic disorders including insulin resistance and type 2 diabetes. The picture is created with BioRender.com.

The IRF3 KO mice develop obesity, IR and T2D spontaneously associated with the development of WAT inflammation. IRF3 regulates the development of obesity and obesity-associated diseases through multiple mechanisms. IRF3 KO mice have less energy expenditure and reduced activity compared to WT mice (Fig. 1g), which result in a positive energy balance and subsequent obesity. During adipocyte differentiation, IRF3 restrains the activation of PPARγ-regulated adipogenic program (Fig. 4). Conversely IRF3 KO pre-adipocytes exhibited earlier and increased expression of lipogenesis program compared to WT cells during differentiation, and had increased lipid droplets once matured (Fig. 3e). In addition, altered expression of genes important for the function of adipocytes, including genes regulating insulin sensitivity [26], inflammatory mediators and their receptor signaling, and PKCδ/PTPN6 pathway was observed in IRF3 KO adipocytes (Fig. 4e and Supplementary Fig. 4). These results suggest that IRF3 is required for maintaining normal functionality of adipocyte in obesity. Furthermore, IRF3 also controls the development of WAT inflammation in obesity. IRF3 KO macrophages exhibited an increased M1 pro-inflammatory profile which could aggravate the impairment of insulin sensitivity (Fig. 5). Bone-marrow reconstitution experiments further demonstrated that both nonhematopoietic and hematopoietic cells contributed to the development of obesity-associated metabolic abnormalities in IRF3 KO mice (Fig. 4g, h and Fig. 6g). These results suggest that IRF3 acts coordinately at different cellular compartments to prevent excessive WAT expansion and dysfunctionality, thereby preventing the development of obesity and T2D (Fig. 7).

In obesity, expanding WAT releases increased amount of FFA which is a physical link between obesity, inflammation, and IR [31]. We found that FFA induced IRF3 activation in macrophages to regulate IFNβ expression (Fig. 6). IFNβ is successfully used in treatment of diseases such as multiple sclerosis with underlying mechanism not well understood [32]. Studies demonstrated that induction of IL-10 is important for the anti-inflammatory property of IFNβ For instance, in macrophages, IFNβ is required for LPS-induced IL-10 production to prevent superinduction of inflammatory cytokines [33]. In *Mycobacterium tuberculosis-*infected macrophages, IFNβ inhibits IL-12 and TNFα production mainly through IL-10 [34]. We show here that IFNβ inhibits the expression of TNFα, IL-6, and IL-1β macrophages in response to M1 or FFA mainly through IL-10 (Fig. 6). Neutralizing IFNβ impaired IL-10 expression in WT macrophages leading to increased expression of those cytokines (Fig. 6c, d). Importantly, IFNβ neutralization or exogenous IL-10 abrogated the differences on the expression of those cytokines between WT and KO macrophages upon M1 activation (Fig. 6c-e). Therefore, IFNβ-induced IL-10 is a major mechanism by which IRF3 inhibits macrophage inflammatory activation and adipose tissue inflammation in obesity.

PPARγ is required for M2 macrophage activation [35]. Macrophage PPARγ inhibits IFNβ production by interfering with IRF3-mediated transcription of IFNβ [36]. In addition, deficiency of PPARγ resulted in upregulation of IFNβ to suppress IL-1β and IL-1α [37]. These findings demonstrated that the anti-inflammatory function of PPARγ is possibly through IRF3-IFNβ pathway in macrophages. We observed increased expression of M2 markers in IRF3 KO macrophages (Supplementary Fig. 5a, c). However, unlike in adipocyte differentiation, IRF3 KO macrophages had reduced PPARγ expression with or without stimulation (Supplementary Fig. 6c). These results suggest that IRF3 may regulate PPARγ in a cell type- and context-dependent manner and the anti-inflammatory function of IRF3-IFNβ pathway in macrophages could be independent of PPARγ.

Macrophages are not the only contributor to the development of chronic inflammation in WAT in obesity. T-lymphocytes have also been shown to contribute to chronic adipose inflammation. For instance, obese mice contain higher levels of WAT CD8^+^ T cells that promote macrophage infiltration and adipose tissue inflammation [38]. In addition, adipose tissue from lean animals contains a unique population of regulatory *T* (*T*_reg_) cells that express PPARγ which interacts with Foxp3, and depletion of these Tregs increased the expression of TNFα and IL-6 and adipose tissue inflammation [39, 40]. Complementing with our findings, the anti-inflammatory role of PPARγ documented from this study further substantiates its importance in adipose tissue homeostasis. CD4^+^ T helper cells (Th) such as Th1 cells also play a role in the development of WAT inflammation and IR. For instance, accumulation of CD4^+^ T cells which are Th1 cells in adipose tissue preceded the recruitment of macrophages during the development of obesity and IR in mice [41]. These Th1 cells produce IFNγ to promote the expression of MCP-1 and other inflammatory mediators by pre-adipocytes and adipocytes, thereby facilitating monocyte recruitment and adipose tissue inflammation [41, 42]. While evidences regarding the role of Th2 in adipose inflammation remain limited, a study on human adipose tissue from obese subjects showed an inverse relationship between Th2 frequency and both circulating inflammatory markers and IR [43]. The increased amount of Th17 cells was also observed in visceral adipose tissue in human unhealthy obesity and adipose tissue Th17 cells were believed to contribute to WAT inflammation [44, 45]. IRF3 has been shown to regulate T-cell function. For instance, IRF3 KO T cells had reduced production of IFNγ, IL-17, and Granzyme B upon stimulation [46]. In addition, IRF3 was shown to repress IL-17 production in CD8^+^ T cells through complex formation with RORγt, leading to reduced Th17 polarization [47]. It is important to note that macrophages on its own may also promote the infiltration of T-lymphocytes. For example, resident macrophages in WAT can promote CD4^+^ T-cell activation through antigen presentation, promoting their proliferation, and IFNγ production [48]. Hence, IRF3 could intrinsically regulate accumulation and function of CD4^+^ and CD8^+^ T cells and adipose tissue *T*_reg_ cells, or through macrophages to regulate adipose tissue inflammation, which requires further investigation.

In summary, our study demonstrates that in adipocytes, IRF3 limits adipogenesis by controlling PPARγ signaling and is required for maintaining the functionality of adipocytes. In macrophages, IRF3 suppresses inflammatory activation through the IFNβ/IL-10 axis, thereby inhibiting adipose tissue inflammation during WAT expansion. The function of IRF3 in multiple cellular compartments work cooperatively to prevent the development of obesity-related metabolic abnormalities including IR and T2D (Fig. 7). Further investigation into the mechanisms by which IRF3 regulates various cellular compartments in metabolism will help us to develop novel strategies to target IRF3 for the prevention and treatment of obesity-associated diseases.

## Supplementary information

Supplementary Figures
