# Supplementary material for: Regulation of adipogenic differentiation and adipose tissue inflammation by interferon regulatory factor 3
Source: Cell Death Differ. 2021 Jun 5;28(11):3022–35. doi: 10.1038/s41418-021-00798-9 (PMC8563729; doi:10.1038/s41418-021-00798-9)
Supplement: Supplementary file 1 — Supplementary Figures [file 41418_2021_798_MOESM1_ESM.pdf]

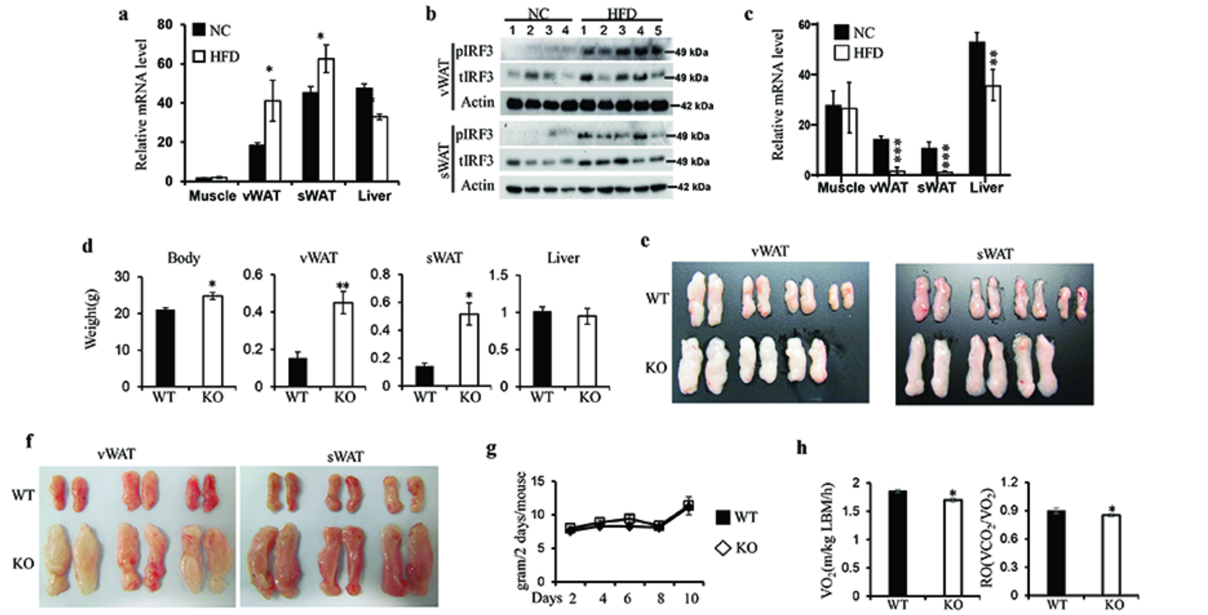

Figure S1. IRF3 regulates the development of obesity in mice. (a-c) Altered expression of IRF3 is associated with obesity. WT mice were fed with a chow diet or a high-fat diet (HFD) for 8 weeks after weaning. IRF3 mRNA levels were detected in skeletal muscle, perigonadal WAT (vWAT), subcutaneous WAT (sWAT) and the liver by quantitative real-time PCR (qPCR). mRNA levels were normalized to GAPDH control (a). (b) Protein expression and phosphorylation of IRF3 in vWAT and sWAT after 8 weeks of HFD were detected by western blot analysis. (**c**) WT mice were fed with a chow diet or a high-fat diet (HFD) for 16 weeks after weaning. IRF3 mRNA expression in various organs and tissues was examined qPCR. (d) The weights of the body, vWAT, sWAT and the liver from 6-month old female WT and IRF3 KO mice were determined. (**d**) Representative pictures of perigonadal and subcutaneous fat pads isolated from 4-month old WT and IRF3 KO male mice. (**e**) Representative pictures of subcutaneous and pre-gonadal fat pads from 6-month old of WT and KO female mice. (**f**) Comparable food intake by WT and IRF3 KO mice. Average food intake by age-matched (3 and 4 months) WT and KO male mice was monitored for 10 days. (**g**) O2 consumption and respiratory exchange ratio of WT and IRF3 KO mice were analyzed by CLAMPs. Data are presented as mean ± SEM. ∗p< 0.05, ∗∗p< 0.01.

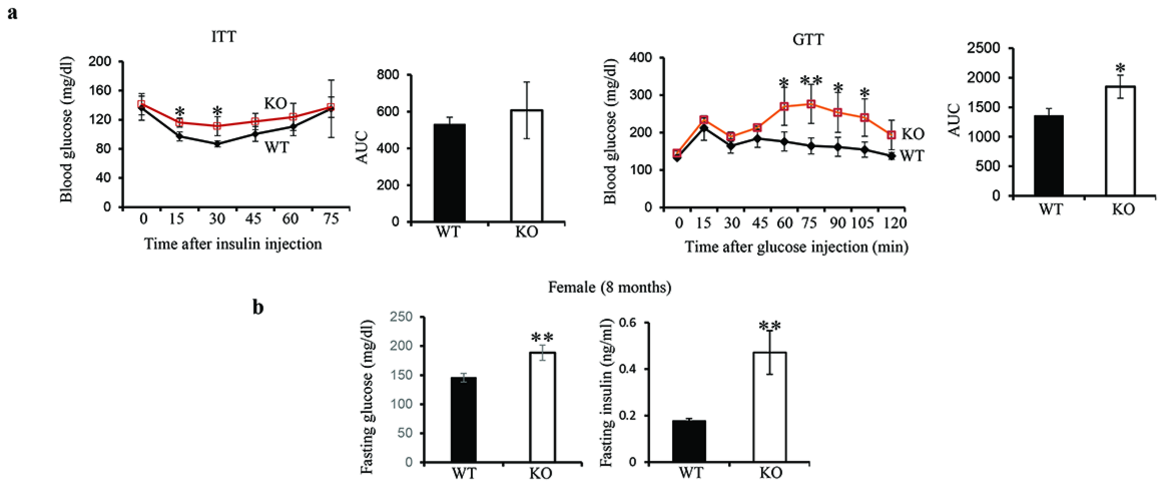

Figure S2. IRF3 deficiency resulted in impaired glucose homeostasis and type 2 diabetes. (**a**) Insulin tolerance tests were performed in female 5-month old WT (n=5) and IRF3 KO mice (n=5) after injection of 0.7U/kg human insulin (Sigma, MO). Glucose tolerance tests were performed on sex-matched WT (n=5) and IRF3 KO mice (n=5) at the same age after they were fasted overnight by giving 2g/kg of D-glucose ip. Blood samples were taken at the indicated time points and blood glucose levels were measured with an Ascensia Elite XL glucose meter and test strips. (b) Fasting blood glucose and serum insulin levels of 8 month old female mice were determined. The data shown are a representative of 3 to 5 independent experiments with similar results. Data are presented as mean ± SEM. *$p < 0.05$, **$p < 0.01$.

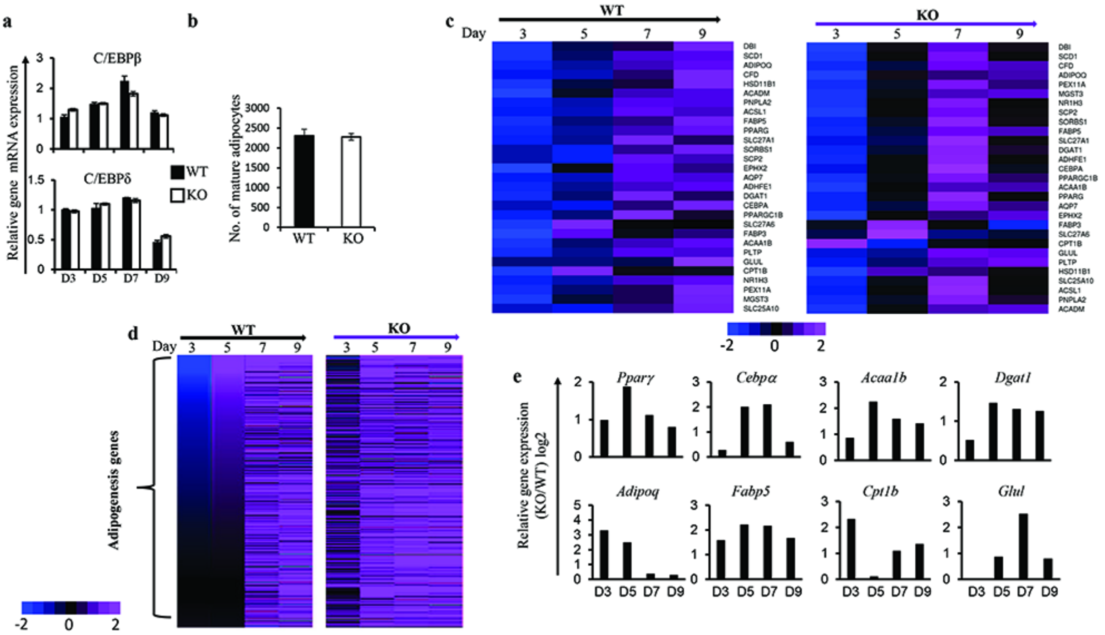

Figure S3. (**a**) Comparable expression of *C/EBPb and C/EBPd* between WT and IRF3 KO cells during adipocyte differentiation. Cells were harvested at various days during differentiation for RNA. The expression of *C/EBPb and C/EBPd* was determined by qPCR. (**b**) Comparable numbers of mature adipocytes between WT and IRF3 KO cells after differentiation. WT and IRF3 KO pre-adipocytes were differentiated in 24 well plates for 11 days. Cells were harvested and total cell numbers in each well were determined. (**c**) Heat map showing sequential up-regulation of PPARγ-targeted gene expression in both WT and IRF3 KO adipocyte during differentiation analyzed by microarray. (d) Heat map showing early and enhanced expression of adipogenic gene expression in IFR3 KO adipocytes compared to WT cells during differentiation. (**e**) Relative expression of PPARγ-targeted genes in WT and KO adipocytes at various days during differentiation.

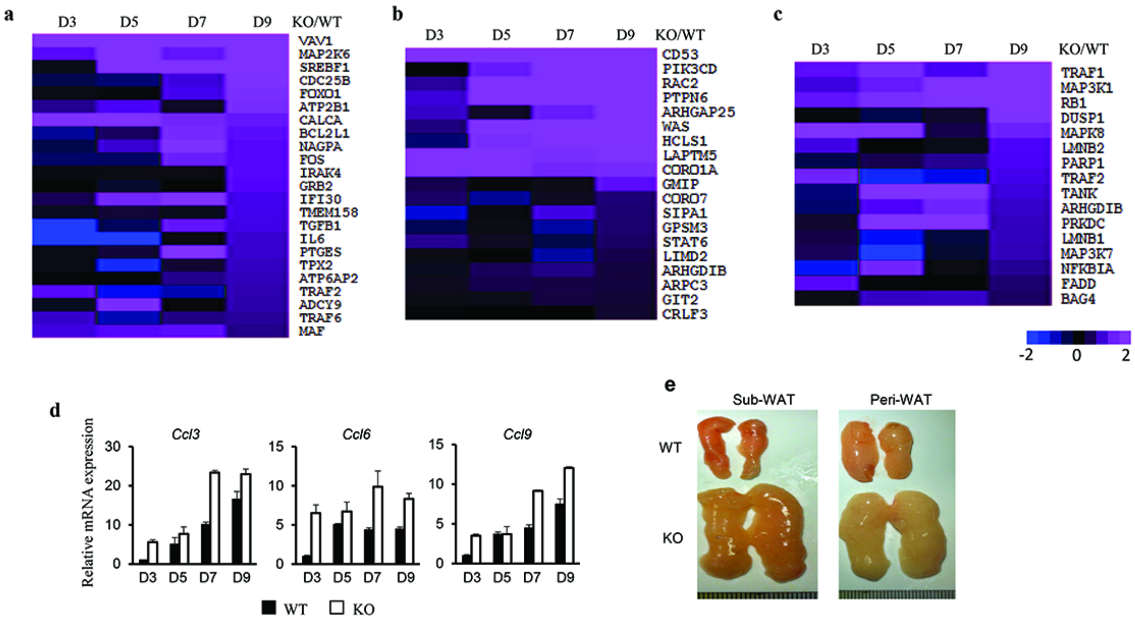

Figure S4. (**a-c**) Heat map showing enriched expression of inflammatory cytokine and receptor signaling genes (a), genes involved in PKCδ/PTPN6 axis (b), and insulin-resistant genes (c) in KO adipocytes over WT cells. (**d**) Expression of *Ccl3*, *Ccl6* and *Ccl9* in WT and KO adipocytes at various days during differentiation was determined by qPCR. (**e**) Representative pictures of subcutaneous and pre-gonadal fat pads from lethally irradiated WT or KO mice at eighteen weeks after they received WT bone marrow (BM) cells.

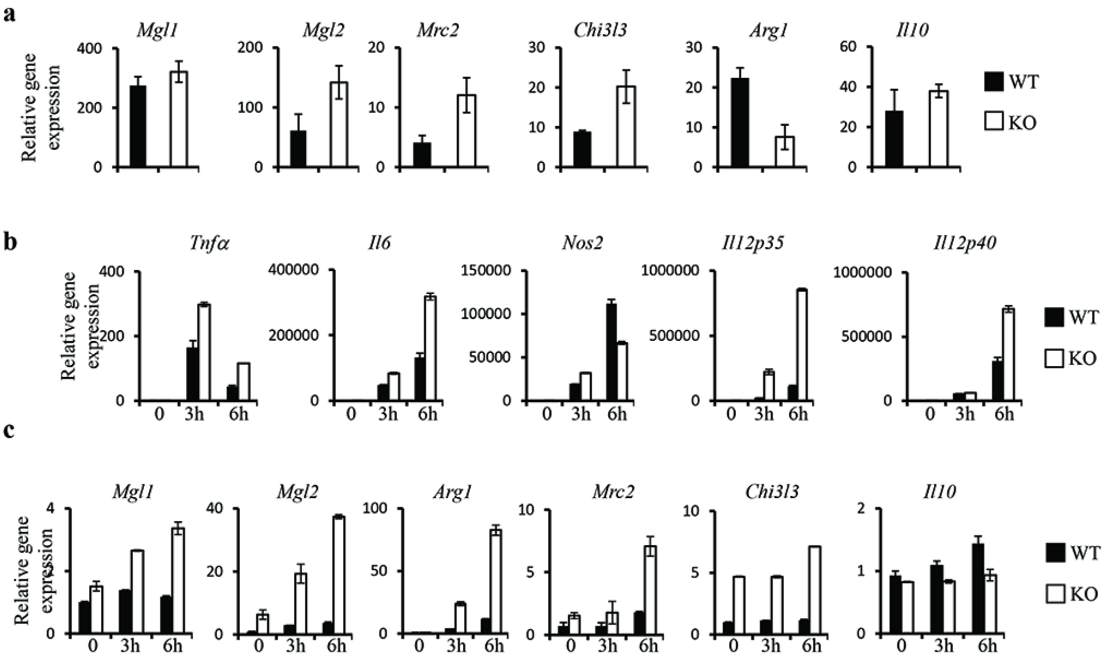

Figure S5. (**a**) Stromal vascular fractions (SVFs) were isolated from WT and IRF3 KO WATs

for RNA. The expression of M2 macrophage genes was determined by qPCR. WT and IRF3 KO bone marrow-derived macrophages (BMDMs) were stimulated with M1 (**b**) or M2 (**c**) activation conditions. The expression of M1 or M2 markers were examined by qPCR, respectively.

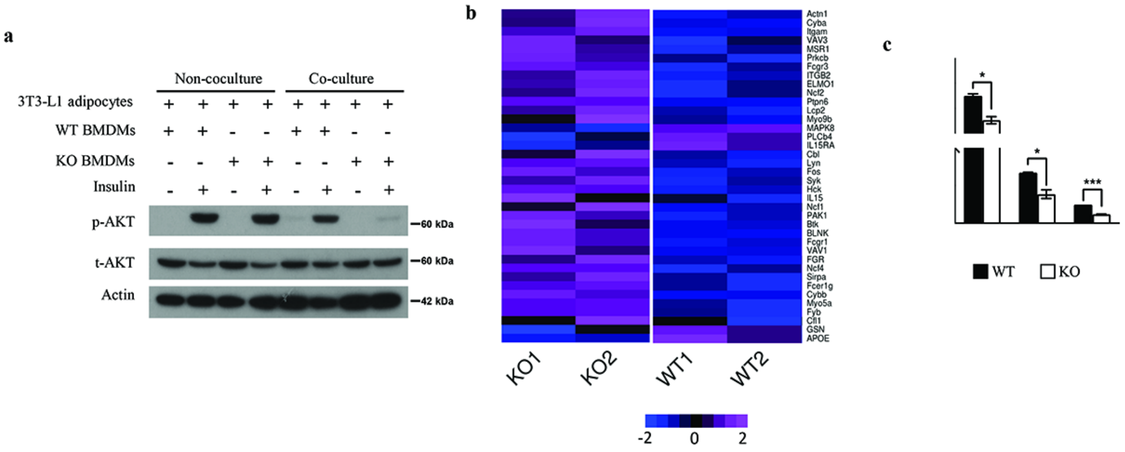

Figure S6. (**a**) Reduced AKT activation in adipocytes cocultured with IRF3 KO macrophages compared to cells cocultured with WT macrophages in response to palmitate treatment. 3T3-L1 pre-adipocytes were differentiated into mature adipocyte before co-cultured with WT or IRF3 KO BMDMs. Cells were stimulated with 500mM palmitate overnight followed by stimulation with 100nM insulin for 15 min to assess AKT activation. (**b**) vWATs were isolated from WT and IRF3 KO mice at the age of 6 months for microarray analysis. Heap map showing increased expression of phagocytic genes in IRF3 KO WAT compared with these in WT WAT. (**c**) WT and IRF3 bone marrow-derived macrophages were stimulated with 100 ng/mL LPS and 20 ng/mL IFNγ for 0, 3, 6 hours to harvest cells for total RNA. The expression of PPARγ was assessed by qPCR.

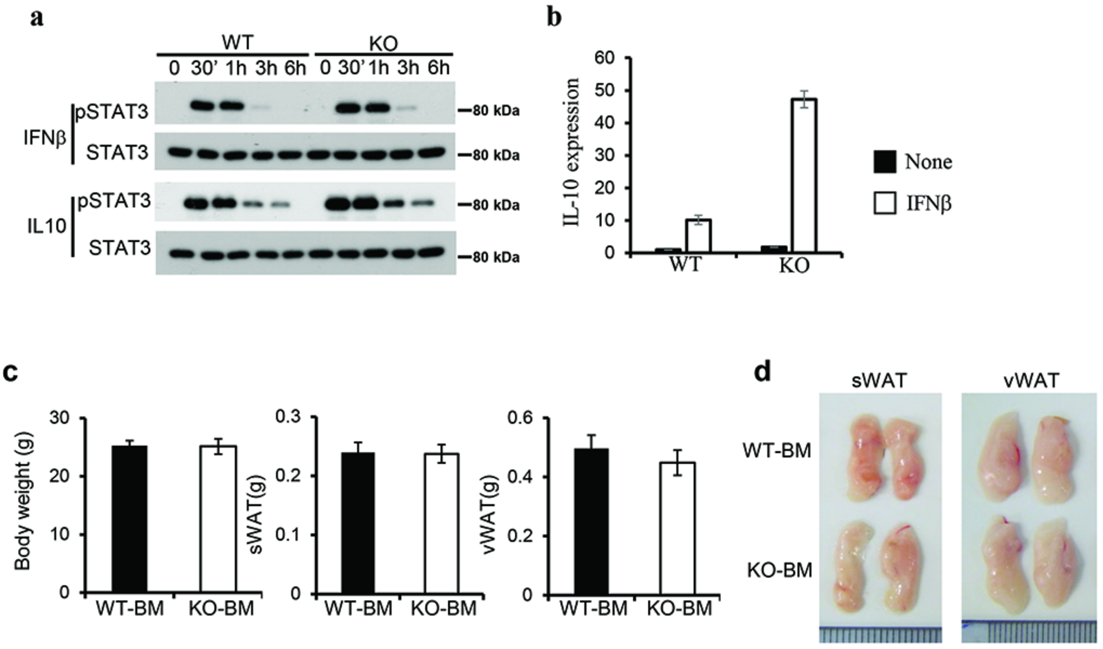

Figure S7. (**a**) WT and IRF3 KO BMDMs were stimulated with IFNβ (2ng/mL) or IL-10 to examine STAT3 phosphorylation by western blot analysis. (**b**) WT and IRF3 KO BMDMs were stimulated with IFNβ (10ng/mL) followed by examination of IL10 expression by qPCR. (**c-d**) Lethally irradiated WT mice were reconstituted with WT (WT-BM) or IRF3 KO BM (KO-BM) cells. Three months after reconstitution, weights of body, sWAT and vWAT were measured (**c**), and represented pictures of sWAT and vWAT from the chimeric mice are presented (**d**).
